# Establishing the 99^th^ percentile for high sensitivity cardiac troponin I in healthy blood donors from Southern Italy

**DOI:** 10.11613/BM.2019.020901

**Published:** 2019-06-15

**Authors:** Luisa Agnello, Chiara Bellia, Concetta Scazzone, Giulia Bivona, Giorgia Iacolino, Caterina Maria Gambino, Maddalena Muratore, Bruna Lo Sasso, Marcello Ciaccio

**Affiliations:** 1 Institute of Clinical Biochemistry, Clinical Molecular Medicine and Laboratory Medicine, Department of Biomedicine, Neurosciences and Advanced Diagnostics, University of Palermo, Palermo, Italy; 2 Unit of Trasfusional Medicine, Villa Sofia-Cervello Hospital, Palermo, Italy; 3 Department of Laboratory Medicine, AOUP “P. Giaccone”, Palermo, Italy

**Keywords:** troponin I, myocardial infarction, reference values, high sensitivity, 99th percentile

## Abstract

**Introduction:**

The knowledge of high sensitivity cardiac troponin I (hsTnI) distribution in a reference population is mandatory for its introduction in clinical practice. The aim of this study was to define the Upper Reference Limit (URL) of hsTnI measured by Single Molecule Counting technology (SMC) in an accurately selected reference population.

**Materials and methods:**

In the study 1140 blood donors were included and selected on the basis of medical history and biomarkers. High sensitivity cardiac troponin I was measured by SMC technology (Clarity, Singulex, Alamed, USA). The 99th percentile was calculated by the non-parametric method according to the Clinical and Laboratory Standard Institute - CLSI C28-A3.

**Results:**

The median age was 41 years (IQR: 28 - 50) and 69% were males. The overall 99th percentile was 5 ng/L (90% CI: 4.2 - 5.6). When considering sex-related differences, we found slight differences between the 99th percentile in males and females. Moreover, the 99th percentile trended with age, especially in females.

**Conclusions:**

We defined the 99th percentile of hs-cTnI measured by SMC technology in a highly selected healthy population, with only minor differences between males and females. Our findings provide the basic criteria for the reliable interpretation of hsTnI concentrations measured by the SMC technology in clinical settings.

## Introduction

Major cardiovascular events still represent a main health issue due to their high mortality and the need of a prompt diagnosis in order to reduce mortality (*1*, *2*). Cardiac troponin I and T (cTnI and cTnT) measurements are the standard of practice in emergency setting supporting the diagnosis of myocardial infarction (MI); assessing prognosis of patients with acute coronary syndrome (ACS); predicting cardiovascular risk in the general population (*3*, *4*).

Basing on the fourth universal definition of myocardial infarction, the upper reference limit (URL) of troponin, defined as the 99^th^ percentile of cTnI distribution in a reference population, has been confirmed as the decision threshold for MI diagnosis (*3*). To date, no universal protocol or guidelines have been drawn up to guide the definition of the 99^th^ percentile for high-sensitivity cardiac troponin and different results have been published using different assays. Among the currently available high-sensitivity (hs-cTnI) assays, the Singulex Clarity cTnI assay measured by Single Molecule Counting (SMC) technology (Singulex, Alamed, USA) represents one of the most sensitive assay detecting very low circulating cTnI concentrations. In a recent cohort study, Kaess *et al*. showed that also a slight increase of cTnI measured by a hs-cTnI assay is an independent predictor of incident coronary heart disease (CHD) in the general population (*4*). Thus, the accurate definition of the 99^th^ percentile of hs-cTnI is of paramount importance.

The aim of this observational study was to define the URL of cTnI by using the SMC technology developed by Singulex in an accurately selected reference population.

## Materials and methods

### Study design

This observational study included 1140 consecutive blood donors recruited from the Unit of Transfusion Medicine of Villa Sofia-Cervello Hospital in Palermo, from October 2017 to February 2018. Health status of blood donors was evaluated through a questionnaire about their past and present health status and lifestyle and by a physical examination (*5*). The inclusion criterion for blood donation was age 18 to 65 years. Exclusion criteria for blood donation were cancer, autoimmune or cardiovascular diseases (*e.g.*, coronary artery disease, angina, cardiac arrhythmias, history of cerebrovascular diseases, arterial thrombosis, recurrent deep vein thrombosis, hypertension with organ damage), organic diseases of the central nervous system (CNS), transplant recipients, diagnosis of haemostatic disorders, epilepsy, anaphylaxis, drug use, chronic alcoholism, infectious diseases and any chronic hepatic, gastrointestinal, urogenital, hematologic, immunologic, renal, metabolic and respiratory disorder. Selected surrogate biomarkers were used to identify clinically asymptomatic diseases: B-type natriuretic peptide (BNP) for myocardial dysfunction (BNP > 35 ng/L); fasting plasma glucose (FPG) for diabetes (FPG > 7 mmol/L); and creatinine for the calculation of the estimate glomerular filtration rate (eGFR) in order to assess chronic kidney disease (eGFR < 60 mL/min/1.73m^2^).

Residual biological material was used for all biochemical analysis and data were anonymised before entering the study so no written informed consent was required from participants. The study was approved by the local Ethic Committee.

### Biochemical analysis

Blood samples were collected in a fasting state. After collection, FPG and serum creatinine was measured immediately by the Architect C800 (Abbott Laboratories, Wiesbaden, Germany). K_2_-EDTA plasma (Greiner Bio-One, Kremsmünster, Austria) was aliquoted and stored at – 80 °C until hs-cTnI and BNP analyses were performed at the end of the enrolment in consecutive sessions using the same lot of reagents.

The concentration of BNP was measured by the Architect BNP assay on Architect i1000 instrument (Abbott Laboratories, Wiesbaden, Germany), which is characterized by a limit of detection (LoD) < 10 ng/L and an imprecision (coefficient of variation (CV %)) < 12%, as declared by manufacturer.

Estimate glomerular filtration rate was calculated by the Chronic Kidney Disease - Epidemiology Collaboration (CKD-EPI) equation.

The concentration of hs-cTnI was measured by Clarity cTnI assay on the Clarity System (Singulex, Alamed, USA), a fully-automated platform based on SMC technology coupled with fluorescent 1-step microparticle-based immunoassays. Briefly, paramagnetic microparticles coated with fluorescently labelled antibodies recognize TnI in plasma. The SMC system detects single fluorescently-labeled molecules by a confocal fluorescence microscope with an avalanche photodiode detector. The limit of quantification (LoQ) corresponding to a CV of 20% was 0.14 ng/L, while the LoD was 0.08 ng/L, as declared by the manufacturer.

### Statistical analysis

Normally distributed variables are presented as mean ± standard deviation (SD), not-normally distributed continuous variables as medians and interquartile ranges (IQR), and categorical variables as percentage. Normality of distributions was assessed using the Kolmogorov-Smirnov test. Differences of hs-cTnI concentration among age and sex groups were evaluated by Kruskal Wallis test. Outliers were detected in the total population using the method of Tukey using both a 1.5 and 3 interquartile ranges as the gating parameter after logarithmic transformation. The 99^th^ percentile of the distribution together with the 90% confidence interval (CI) was calculated after outliers removal using the non-parametric percentile method, in accordance with the Clinical and Laboratory Standard Institute - CLSI C28-A3 (*6*). For statistical analysis, the value of 0.14 ng/L was assigned to hs-cTnI concentrations lower than the limit of quantification.

## Results

**Figure 1:**
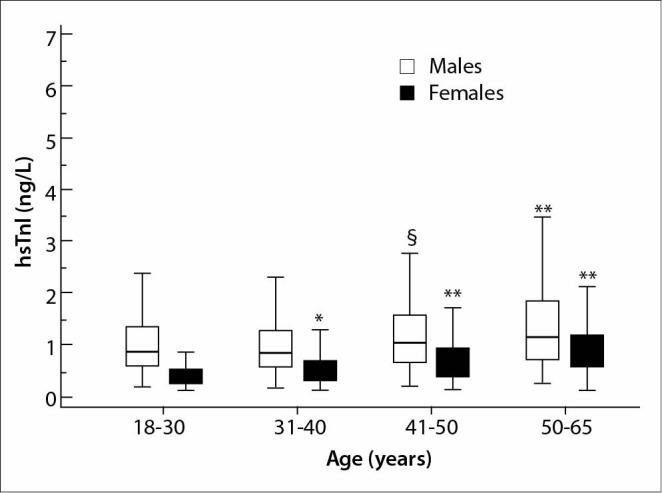
High sensitivity cardiac troponin I plasma concentrations according to age in males and females. *P = 0.003; **P < 0.001; ^§^P = 0.011 *vs*. subjects aged 18-30 years.

The study population comprised 1140 Caucasian individuals from South Italy. After the exclusion of subjects with serum BNP > 35 ng/L (N = 30), 1110 individuals were included in the analysis. No subjects with eGFR < 60 mL/min and FPG > 7 mmol/L were detected. Notably, hs-cTnI was measurable in the 99% (N = 1099) of the study population and it was not-normally distributed. The median age was 41 years (IQR: 28 – 50), with values ranging from 18 to 64 years, and 69% were males. Median hs-cTnI concentration was 0.8 ng/L (IQR: 0.5–1.4). Overall, males had higher concentrations of hs-cTnI than females [1.0 (0.7–1.6) ng/L *vs.* 0.5 (0.3–0.9) ng/L; P < 0.001], and this trend was observed also when subjects were stratified according to age (Figure 1). In particular, in males, plasma hs-cTnI was 0.9 (0.6–1.3) ng/L, 0.8 (0.6–1.3) ng/L, 1.0 (0.7–1.6) ng/L, 1.2 (0.8–1.9) ng/L according to increasing age (18-30, 31-40, 41-50, 50-65 years, respectively). In females, plasma hs-cTnI was 0.3 (0.2–0.5) ng/L, 0.4 (0.3–0.4) ng/L, 0.6 (0.4–0.9) ng/L, 0.8 (0.6–1.2) ng/L according to increasing age (Figure 1). Overall hsTnI 99^th^ percentile was 5 ng/L (90% CI: 4.2–5.6). Given the differences of hs-cTnI between males and females and the statistically significant trend with increasing ages, the 99^th^ percentile of hs-cTnI was calculated also in males and females separately according to age (Table 1). Specifically, 99^th^ percentile of hsTnI was slightly lower in females than in males independently by age.

**Table 1:** 99^th^ percentile of the distribution of hs-cTnI in males and females according to age.

	**N (%) of outliers**	**N**	**99^th^ percentile (ng/L)**	**90% CI**
**Males** **≤ 40 years** **> 40 years**	6 (1.8) 9 (2)	318 432	5.0 5.2	3.8-6.1 4.5-6.1
**Females** **≤ 40 years** **> 40 years**	7 (3.5) 2 (1.4)	191 144	4.3 4.6	3-5.6 3.3-6
**Total**	24 (2.1)	1086	5.0	4.2-5.6
N - number. CI – confidence interval.				

## Discussion

In this observational study the 99^th^ percentile of plasma hs-cTnI was defined in blood donors strictly selected by surrogate biomarkers. The most relevant result of our study is that sex-related differences were modest. Although the use of sex-specific cut-offs has been recommended when using most of the available hs-cTnI assays, these findings demonstrated that the sex-related differences of hs-cTnI concentrations are slight when measured by SMC technology. Our results are in accordance with previous studies performed using the same technology (*7*). Garcia-Osuna showed that the 99^th^ percentile of hs-cTnI measured by Singulex Clarity cTnI assay was 7.12 ng/L and 8.97 ng/L in females and males, respectively. Although this sex-related difference was statistically significant, the clinical relevance of these differences has to be documented. Similarly, Estis *et al.* documented that the 99^th^ percentile of hs-cTnI measured by Singulex Erenna cTnI assay was 6.1 ng/L and 5.8 ng/L in males and females older than 50 years, respectively (*8*).

Although several reports documented a higher diagnostic accuracy of hs-cTnI when sex-specific cut-offs are used; the clinical consequences in terms of cardiovascular outcomes and mortality of using a unique hsTnI cut-off assessed by SMC technology haven’t been addressed yet (*9*). Thus, the clinical evaluation of a sex-specific hs-cTnI cut-off in comparison to a unique one is mandatory when this assay is used.

The distribution of hsTnI should be described accurately given the relevant clinical consequences of an incorrect URL. The International Federation of Clinical Chemistry (IFCC) Task Force on Clinical Applications of Cardiac Biomarkers provided some recommendations to enrol presumably healthy individuals but only a small number of studies have been performed accordingly (*10*). Moreover, regarding the Singulex Clarity cTnI assay, only one study including blood donors has been published (*7*). However, the Authors studied a modestly sized and ethnically heterogeneous population, finding a higher 99^th^ percentile than the our [5 ng/L (90% CI: 4.2–5.6) *vs*. 8.01 ng/L (95% CI: 6.01-10.36)]. The added value of our study was the enrolment of a larger, ethnically homogeneous sample, including 1110 individuals from Southern Italy.

The high sensitivity of Singulex Clarity hs-cTnI assay makes it a good candidate for rule-out/rule-in strategies of ACS in Emergency setting as well as the stratification of the risk of major cardiovascular events in patients with suspected CHD. However, the introduction of this assay in the current format into clinical practice is limited by some factors, including a long sample processing time (40 min) and the absence of an adequate management of STAT requests.

In the present study, no comparison with other available hs-cTnI assays and no evaluation of the imprecision at the 99^th^ percentile were performed. Moreover, no individuals aged > 65 years were included in our study population. This might have contributed to an underestimation of 99^th^ percentile. Nevertheless, blood donors that represent the most likely healthy individuals can be considered an ideal population for accurately estimating the reference limits of laboratory parameters.

In conclusion, our findings provide the basic criteria for the clinical interpretation of hs-cTnI measured by the SMC technology.
